# Developing Carrier Complexes for “Caged NO”: RuCl_3_(NO)(H_2_O)_2_ Complexes of Dipyridylamine, (dpaH), N,N,N'N'-Tetrakis (2-Pyridyl) Adipamide, (tpada), and (2-Pyridylmethyl) Iminodiacetate, (pida^2-^)

**DOI:** 10.1155/MBD.2000.67

**Published:** 2000

**Authors:** Joseph M. Slocik, Richard A. Kortes, Rex E. Shepherd

**Affiliations:** Department of Chemistry University of Pittsburgh Pittsburgh Pennsylvania 15260 USA

## Abstract

Delivery agents which can carry the {Ru(NO)}^6^ chromophore (“caged NO”) are desired for vasodilation and for photodynamic therapy of tumors. Toward these goals, complexes derived from [RuCl_3_(NO)(H_2_O)_2_]= (**1**) have been prepared using dipyridylamine (dpaH) as mono and bis adducts, [Ru(NO)Cl_3_(dpaH)] = (**2**) and [Ru(NO)Cl(dpaH)_2_]Cl_2_ = (**3**). The dpaH ligands coordinate cis to the Ru(NO) axis.The mono derivative is a model for a potential DNA groove-spanning binuclear complex {[RuNO)Cl_3_]_2_(tpada)} = (**4**) which has two DNA-coordinating Ru^II^ centers, photo-labile {Ru(NO)}^6^ sites, and a groove-spanning tether moiety.The binuclear assembly is prepared from the tethered dipyridylamine ligand N,N,N',N'-tetrakis(2-pyridylmethyl)adipamide (tpada) which has recently been shown to provide a binuclear carrier complex suited to transporting Ru^II^ and Pd^II^ agents. A related complex, [Ru(NO)Cl(pida)] = (**5**) with the {Ru(NO)}^6^ moiety bound to (2-pyridylmethyl) iminodiacetate (pida^2-^) is also characterized as a potential “caged NO” carrier. Structural information concerning the placement of the pyridyl donor groups relative to the {Ru(NO)}^6^ unit has been obtained from ^1^H and ^13^C NMR and infrared methods, noting that a pyridyl donor trans to NO+ causes “trans strengthening” of this ligand for [Ru(NO)Cl(pida)], whereas placement of pyridyl groups cis to NO+ causes a weakening of the N-O bond and a lower NO stretching frequency in the dpa-based complexes.


### DEVELOPING CARRIER COMPLEXES FOR "CAGED NO": $RuCl_3(NO)(H_2O)_2$ COMPLEXES OF DIPYRIDYLAMINE, (dpaH), N,N,N'N'-TETRAKIS(2-PYRIDYL)ADIPAMIDE, (tpada), AND (2-PYRIDYLMETHYL)IMINODIACETATE, ($pida^{2-}$)

Joseph M. Slocik, Richard A. Kortes and Rex E. Shepherd*

Department of Chemistry, University of Pittsburgh, Pittsburgh, Pennsylvania 15260, USA

### Abstract

Delivery agents which can carry the $\{Ru(NO)\}^6$ chromophore (" caged NO ") are desired for vasodilation and for photodynamic therapy of tumors. Toward these goals, complexes derived from $[RuCl_3(NO)(H_2O)_2]$ = (**1**) have been prepared using dipyridylamine (dpaH) as mono and bis adducts, $[Ru(NO)Cl_3(dpaH)]$ = (**2**) and $[Ru(NO)Cl(dpaH)_2]Cl_2$ = (**3**). The dpaH ligands coordinate cis to the Ru(NO) axis.The mono derivative is a model for a potential DNA groove-spanning binuclear complex $\{[Ru(NO)Cl_3]_2(tpada)\}$ = (**4**) which has two DNA-coordinating $Ru^{II}$ centers, photo-labile $\{Ru(NO)\}^6$ sites, and a groove-spanning tether moiety.The binuclear assembly is prepared from the tethered dipyridylamine ligand N,N,N',N'-tetrakis(2-pyridylmethyl)adipamide (tpada) which has recently been shown to provide a binuclear carrier complex suited to transporting $Ru^{II}$ and $Pd^{II}$ agents. A related complex, [Ru(NO)Cl(pida)] = (**5**) with the $\{Ru(NO)\}^6$ moiety bound to (2-pyridylmethyl)iminodiacetate ($pida^{2-}$) is also characterized as a potential "caged NO" carrier. Structural information concerning the placement of the pyridyl donor groups relative to the $\{Ru(NO)\}^6$ unit has been obtained from $^1H$ and $^{13}C$ NMR and infrared methods, noting that a pyridyl donor trans to $NO^+$ causes "trans strengthening" of this ligand for [Ru(NO)Cl(pida)], whereas placement of pyridyl groups cis to $NO^+$ causes a weakening of the N-O bond and a lower NO stretching frequency in the dpa-based complexes.

### Introduction

The complex $[RuCl_3(NO)(H_2O)_2]$ = (**1**) has been studied by Bettache, Carter et al. as a source of "caged NO ", a reagent suited to vasodilation and photodynamic therapy [1,2]. Other photo-releasing systems for NO dissociation have been reviewed [3,4]. The quantum yield for photolysis of (1) is low ($\Phi = 0.012$] [1], requiring the development of other ligand donors that can enhance the photo-dissociation chemistry in order to have a viable agent for photodynamic therapy. Additionally, the biodistribution of (1) has not been studied. Information on what physiologically accessible donors bind to the $\{Ru(NO)\}^6$ core have been unavailable except for the report of enhanced electrophoretic mobility in the presence of glycine or alanine [5]. $RuCl_3(NO)$-derived species are also reported to be the ones involved in the transport of radioactive $^{106}Ru$ into fish and other aquatic species [5].

Well characterized complexes derived from $RuCl_3(NO)$ have only been reported in 1999. A tridentate methionine complex $[Ru(NO)Cl_2(methionine)]$ complex that has been characterized by X-ray diffraction [6]. Only recently have the complexes of (**1**) with imidazole at 1 : 1, 1 : 2 and 1 : 3 ratio of $\{Ru(NO)\}^6$ : imidazole, and 1 : 1 complexes with histidine and histamine been reported [7]. The main species of these imidazole, histidine and histamine complexes are as shown in drawings 1 -5 on the next page. Imidazole addition occurs cis to the $NO^+$ moiety at 1 : 1 and 1 : 2, followed by trans addition at 1 :3. However, histidine addition at 1 : 1 has the imidazole donor trans to $NO^+$ due to an electrostatic steering from the anionic carboxylate to $NO^+$ that directs the imidazole donor in the trans location. The 1 : 1 histamine complex follows the cis addition of imidazole since it lacks a directing carboxylate group.

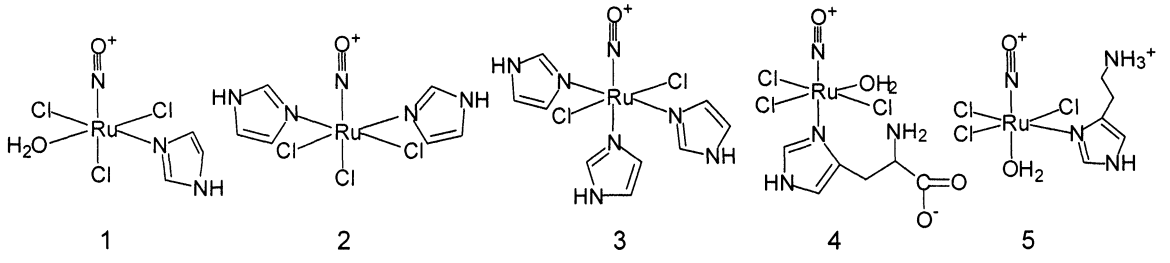

In another current report from our research group [8], we have described the coordination of the $\{Ru(NO)\}^6$ unit to the peptides (gly-gly-gly-2H) and (gly-gly-his-2H) wherein the peptide adopts a planar coordination with an axial {Ru(NO)} group. Attachment is via the terminal amino donor, two peptido ionized N donors and $Cl^-$ for (gly-gly-gly-2H) or the imidazole donor for (gly-gly-his-2H) [8]. The behavior of the $\{Ru(NO)\}^{3+}$ unit toward peptide coordiation is the same as the well-characterized square-planar analogues of $Pd^{II}$, $Pt^{II}$ and $Ni^{II}$, except that the $\{Ru(NO)\}^{3+}$ complexes have the axial $NO^+$ and $Cl^-$ or $H_2O$ donors, whereas the $Ni^{II}$, $Pd^{II}$, $Pt^{II}$ family are strictly square-planar.

The present paper describes stable complexes that can be obtained using pyridyl-based donors for the $\{Ru(NO)\}^6$ core. Godwin and Meyer have prepared a bis bipyridine complex, $[Ru(NO)Cl(bpy)_2]^{2+}$, which by virtue of steric constraints from the two bpy ligands cannot retain the four pyridyl donors in the plane cis to $NO^+$ [9]. A pseudo-square planar arrangement of ligands has an appeal for the development of carrier complexes of NO that might associate with DNA by intercalation as a means of bringing "caged NO" into the environment of DNA. Photolysis of such bound "caged NO" species could then be used to induce local DNA cleavage. Our efforts in this regard have involved the use of the dipyridylamine (dpaH) ligand, which due to the central nitrogen linkage, affords a flexible donor that avoids the steric problems associated with two bipyridines. Indeed, it is reported herein that 1 : 1 and 1 : 2 complexes with dpaH are obtained with ligands cis to $NO^+$.

An extension of the simple dipyridylamine donor in which two dipyridylamine moieties are linked by an adipyl chain forming the recently prepared ligand tpada [10], has been carried out to incorporate two $RuCl_3(NO)$ headgroups that are bound by tpada. This arrangement affords a "caged NO" binuclear complex that in principal can span the major groove of DNA, form local coordination adducts by nucleobase displacement of $Cl^-$, and can stand ready for the photo-release of NO.

Lastly, the present paper describes the coordination of the pyridyl-aminocarboxylate hybrid ligand (2-pyridylmethyl)iminodiacetate, ($pida^{2-}$). NMR evidence indicates a major product [Ru(NO)Cl(pida)] with the pyridyl donor trans to $NO^+$. Such modifications in which the donor properties of the group trans to $NO^+$ has promise as a route to enhancement of the photo-lability of the NO chromophore. The {Ru(NO)Cl(pida)] complex may be one of a series of trans-modified, "caged NO" complexes of use to photodynamic therapy.

### Materials and Methods

Reagents: $[RuCl_3(NO)(H_2O)_2$ was prepared by the method of Fletcher et al. [11] from $RuCl_3(H_2O)_3$ (Aldrich). 2,2'-dipyridylamine was also used as supplied by Aldrich. (2-pyridylmethyl)iminodiacetic acid ($H_2$pida) synthesized for a former study of the [Fe(NO)(pida)] [12] and used from that supply. N,N,N'N'-tetrakis(2-pyridyl)adipamide (tpada) was synthesized as described in a recent publication [10]. All other reagents were analytical grade. Water was distilled deionized water from a house supply.

Instrumentation: $^1H$ and $^{13}C$ NMR spectra were recorded on a 300 AC Bruker NMR spectrometer. Solid samples were dissolved in $D_2O$ (Aldrich) and calibrated against the 4.80 ppm HOD resonance line. pD adjustments were made using NaOD or DCl. $^{13}C$ NMR spectra were calibrated against p-dioxane (66.6 ppm) in the proton decoupled mode. Signals were averaged for $^{13}C$ NMR for a minimium of 8 h. Typical techniques matched those of recent prior publications on N-heterocyclic and $NO^+$ complexes of $[Ru^{II}(hedta)]^-$ [13-15]. UV-visible spectra were recorded on a Varian-Cary 118C scanning spectrophotometer using 1.00 and 0.100 cm quartz cells, and maintaining the concentrations of the complexes in the range of $1.00x10^{-3}$ to $2.00x10^{-2}$ M by weighing out samples on an analytical balance and dissolving the solids in volumetric flasks. Infrared spectra were obtained in the FTIR mode on a ATI Mattson Genesis Series FTIR. Samples were prepared as KBr pellets, pressed at nine tons.

$[Ru(NO)Cl_3(dpaH)]$ (**2**) and $[Ru(NO)Cl(dpaH)_2]Cl_2$ (**3**) samples : 0.2507 g ($1.06x10^{-3}$mol) $RuCl_3(NO)(H_2O)_2$ and 0.1779 g dpaH ($1.04x10^{-3}$ mol) were weighed into a 50 mL round-bottom flask containing a rice sized stirring bar. The flask was sealed with a septum, and then purged with $N_2$ through a syringe needle inlet and exit for 15 min. This was necessary to prevent a catalyzed oxidation of dpaH as determined from a trial without $N_2$ purging. 25.0 mLof $N_2$-purged $H_2O$ was added by a syringe. The sample flask was heated for 2h at near boiling on a water bath while magnetic stirring was achieved. The solution's color changed from purple of (**1**) to a brownish-orange product. A small amount of undissolved dpaH was removed by filtration. The sample was concentrated by rotary evaporation at 45°C. A light tan solid formed and was isolated, washed with cold $H_2O$ and ethanol, and dried in a vacuum desiccator with pumping overnight. The procedure was repeated for the 1 :2 complex, isolated as $[Ru(NO)Cl(dpaH)_2]Cl_2$ by combining 0.2081 g ($8.76x10^{-4}$ mol of (**1**) and 0.3454 g of dpaH ($2.02x10^{-3}$ mol) in 35 mL of $N_2$-purged water in an $N_2$-purged round-bottom flask. The sample cycle included heating for 2h, removal of a trace of unreacted dpaH by filtration, and concentration of the filtrate at 45°C under reduced pressure, followed by drying in the desiccator under vacuum. NMR and IR methods described in the main text establish that the products have high puity, and are absent of unreacted (**1**) or free dpaH.

$\{[RuCl_3(NO)]_2(tpada)\}$ (**4**) : 0.080 g of (**1**) ($3.37x10^{-4}$ mol) and 0.078 g ($1.72x10^{-4}$ mol) of tpada were combined in an empty 50 mL round-bottom flask. After $N_2$ purging of the flask, 20.0 mL of $N_2$-purged deionized water was added by syringe. The flask was heated on a water bath to near boiling for 4.5 h. At the end of this period, there was a small amount of unreacted tpada still visible. The solution color was brownish-orange. The solution was allowed to stir overnight. At that point, a yellowish-brown solid was obtained upon concentration in a rotary evaporator at 45°C under reduced pressure. The final product upon drying had a shiny brown appearance. NMR data showed the absence of unreacted free ligand or the presence of any significant amount of a 1 : 1 adduct that would lower the symmetry of protons along the tether chain. IR data show an absence of unreacted (**1**). Thus, a sample of high purity for a binuclear product is indicated.

[Ru(NO)Cl(pida)] (**5**) : 0.1518 g of (**1**) ($6.39x10^{-4}$ mol) and 0.1425 g of $H_2$pida ($6.36x10^{-4}$ mol) were placed in a 50 mL round-bottom flask with a stir bar, sealed and purged with $N_2$ for 15 min, and 30.0 mL of $N_2$-purged deionized water was added. The solution was heated at 55 to 60°C for 3 h while the solution color slowly changed from the purple of (**1**) to an orange color of the product. This solution was rotary evaporated to a solid and dried under vacuum in a desiccator. NMR indicated a high purity without the presence of unreacted $H_2$pida ligand.

### Results

### *1 : 1 and 1 : 2 dpaH complexes (2) and (3) prepared from (1)*

2,2'-dipyridylamine in $D_2O$ or acidified by DCl show three resonances that integrate 1 : 1 : 2 for the protons. The numbering scheme for the dpaH ligand is given at the top of the next page in a drawing 6 of dpaH. These are are assigned to the H-6,6' pair at 8.30 ppm (indistinguishable doublet), H-4,4' pair at 8.00 ppm (triplet splitting), and an overlapped multiplet at 7.20 ppm for the composite resonances of the H-3,3' and H-5,5' hydrogens.

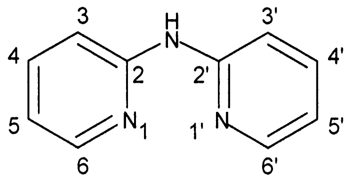

6

Upon coordination in the $[Ru(NO)Cl_3(dpaH)]$ product (**2**), all four protons are differentiated and shifted relative to the free ligand. The H-6,6' pair moves upfield to 8.18 ppm (singlet) ; H-4,4' moves upfield to 7.76 ppm (triplet) while H-3,3' moves downfield to 7.27 ppm (doublet) and H-5,5' shifts upfield to 7.03 ppm as an unresolved singlet. The presence of four resonances indicates the non-lability of the coordinated dpaH in contrast to the observations of lability for coordinated imidazole donors [7]. Also, there is only one set of ligand resonances which affirm that the pyridyl donors of dpaH must be placed symmetrically, and hence, cis to $NO^+$, in the [Ru(NO)Cl(dpaH)] complex. There was no detectable change in the $^1$H NMR spectrum with time for over several days, which rules out a facile isomerization in the coordination mode of dpaH in the complex. Therefore the $Cl^-$ ligands occupy the *fac* positions in the 1 : 1 dpaH complex, and the dpaH ligand does not readily dissociate.

The presence of the nitrosyl group is confirmed by the FT-IR spectrum, showing $\upsilon_{NO}$ at 1850 $cm^{-1}$. The significance of this value in confirming a cis-coordinated dpaH ligand will be discussed later.

The UV-visible spectrum exhibits a band at 430 nm with an ε of 61 $M^{-1}cm^{-1}$, comparable with similar complexes such as $[Ru(NO)Cl(cyclam)](PF_6)_2$ ($\lambda_{max}$ = 435 nm, ε = 54 $M^{-1}$ $cm^{-1}$) [16].

The $^{13}C$ NMR *spectra confirm the* $^1H$ *NMR results, establishing coordination* of dpaH as a bidentate donor. In the free ligand dpaH, the carbon resonances appear at 150.75 ppm for C-2,2', 113.96 ppm for C-3,3', 140.40 ppm for C-4,4', 118.14 for C-5,5' and 142.33 ppm for C-6,6'. In the isolated complex, $[Ru(NO)Cl_3(dpaH)]$ these resonances remain pairwise equivalent, and are shifted 1.00 ppm downfield for C-2,2' to 151.75 ppm and 1.03 ppm downfield for C-4,4' at 141.43 ppm. The other carbons shift upfield : C-6,6' to 141.92 ppm (a -0.41 shift), C-5,5' at 117.81 (a -0.33 ppm shift) and C-3,3' at 113.74 (a -0.22ppm shift). It is noted here that a protonation equilibrium would cause a downfield shift for C-6,6' rather than the observed upfield shift due to the combined influence of the $Ru(Cl_3(NO)$ moiety. That the C-2,2' and C-6,6' resonances are the most strongly affected is another indicator of coordination. The equivalence of the pyridyl rings again requires that one of the rings cannot occupy the position trans to $NO^+$ as this would differentiate the rings.

The complex that is isolated at 1 : 2 $\{Ru(NO)\}^6$ : dpaH stoichiometry yielded a nitrosyl stretching frequency of 1850 $cm^{-1}$. The UV-visible spectrum is nearly the same as for the 1 : 1 complex except that the absorbance maximum is shifted by 5 nm to 435 nm, and the extinction coefficient is modestly higher at 86 $M^{-1}cm^{-1}$. The $^1H$ NMR spectrum of $[Ru(NO)Cl(dpaH)_2]^{2+}$ (**3**) in $D_2O$ has slightly different shifts than for the 1 : 1 complex. However, the important feature is that all four pyridyl donors retain equivalency, requiring that both dpaH ligands are coordinated cis to $NO^+$ and trans to each other. H-6,6' appears at 8.19 ppm as a singlet (only 0.01 ppm downfield of the 1 : 1 complex), the triplet for H-4,4' appears at 7.87 (0.11 ppm downfield of the 1 :1 complex), H-3,3' at 7.22 as an unresolved singlet 0.05ppm *upfield* of the 1 : 1 complex and H-5,5' at 7.11ppm, again *upfield* of the 1 :1 complex. Thus, the detection of two resonances downfield and two resonances upfield of those for the 1 : 1 complex distinguish the 1 : 2 complex as a distinct entity inspite of the identical $\upsilon_{NO}$ value and very similar UV-visible spectra for the 1 : 1 and 1 : 2 complexes. Since the origin of the band near 430 nm is considered to be a spin-forbidden d-d transition ($^1A$ ---> $^3T$) along with partial MLCT mixing of the $Ru^{II}$ ---> $NO^+$ charge transfer [16], it is not surprising that trading cis ligands to the $\{Ru(NO)\}^6$ unit will produce very modest changes in the spectral maximum or probability of the transition. Thus, the net ligand field from cis donors of two $Cl^-$ plus a dpaH ligand may not be so different from two cis dpaH ligands, whereas changing the ligand trans to $NO^+$ would produce a more dramatic change in the visible transition energy.

The $^{13}C$ NMR spectrum of the $[Ru(NO)Cl(dpaH)_2]Cl_2$ complex (**3**) again shows the C-2,2' carbon resonances as the most downfield, shifted 1.64 ppm to 152.39 ppm. The C-4,4' resonance shifts 140.76 ppm (0.36 ppm downfield of the free dpaH ligand), a lesser shift than for the 1 : 1 complex, but in the same electronic sense. The C-6,6' resonances appear upfield at 140.50 ppm, shifted by 1.17 ppm downfield for the 1 : 2 complex instead of an upfield -0.41 ppm shift for the 1 : 1 complex. The origin of this difference is either the differing influence of trans $Cl^-$ donors in the 1 : 1 complex vs. the trans N donors in the 1 :2 complex, or it reflects the flexing motion of the dpaH rings that must occur to produce four NMR-equivalent pyridyl rings in the 1 : 2 complex. Models show that the H-6,6' and C-6,6' atoms may stay below the plane, away from the $\{Ru(NO)\}^6$ moiety. But in the 1 : 2 complex, each H-6,6' proton and their C-6,6' carbons must spend half of their time above the $RuN_4$ plane. This places the H-6,6' and C-6,6' atoms above the axial current in the $NO^+$ triple bond ---- a greatly different environment than for the 1 : 1 complex where the dpaH ligand con maintain these same atoms away from the $\{Ru(NO)\}^6$ unit for a much higher percentage of the time. This gives added evidence for the distinct identies of the 1 : 1 and 1 : 2 complexes. As for the 1 :1 complex, the more electronically remote C-3,3' and C-5,5' resonances move upfield : to 113.54 (a -0.42 ppm shift vs. free L) for C-3,3' and to 117.71 (-0.43 ppm) for C-5,5' carbons. These are only slightly different from the respective -0.22 ppm and -0.33 ppm shifts for the 1 : 1 complex's resonances in the same position. Thus, the large *reversal* for the C-6,6' pair is clear evidence for a unique 1 : 2, eg. $[Ru(NO)Cl(dpaH)_2]^{2+}$ species vs. the 1 : 1 $[RuCl_3(NO)(dpaH)]$ complex.

### *tpada binuclear complex (4) prepared from two equivalents of (1)*

The $^1H$ NMR spectrum of tpada exhibits a doublet at 8.56 ppm for the H-6,6' pair, a triplet at 8.22 ppm for the H-4,4' pair a triplet at 7.69 ppm for H-3,3' and a doublet at 7.36 for the H-5,5' pair and methylene $CH_2$'s at 2.22 ppm for the $CH_2$'s nearer the amide linkage of the dipyridylamine headgroups and 1.52 ppm for the inner pair of $CH_2$'s in the tether chain. The numbering for the tpapda ligand is shown by drawing 7:

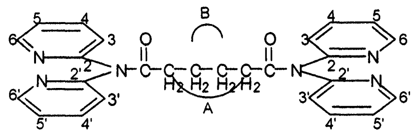

7

Upon coordination to the $RuCl_3(NO)$ moiety at each end of the tpada ligand, one obtains a $^1H$ NMR spectrum showing *differentiated H-6 and H-6' protons* with singlet resonances at 8.43 and 8.27 ppm. H-4,4' appear as an equivalent singlet at 8.01 ppm, H-3,3' has shifted upfield to 7.47 ppm along with H-5,5' to 7.23 ppm. All peaks are rather sharp singlets, but these have lost resolution of their couplings across the ring upon metallation. The tether protons experience downfield shifts to 2.38 ppm for the $CH_2$'s adjacent to the amide linkage and to 1.60 ppm for the interior $CH_2$'s, relatively small shifts as expected by the increasingly remote positions from the $RuCl_3(NO)$ headgroups. The resonance at 2.38 ppm of the $CH_2$'s that are adjacent to the amide linkage are split into a doublet-of-doublets pattern, indicative of a loss of free rotation at this position. This is logically explained by the bulky [$RuCl_3$(NO)(dpa-R)] headgroup that cannot easily rotate in space with motions that randomize orientations as easily as in the free ligand tpada. Allowing for the $\pi$-conjugation that extends from the ruthenated pyridyl donors through the amide linkage --- all attempting to retain planarity to maximize the amido bonding in the carbonyl region-- provides differentiation of the $CH_2$protons which stay fixed in space relative to the orientation of the {Ru(NO)} axis.

This same $\pi$-conjugation around the amide carbonyl establishes planarity of the carbonyl unit together with the pyridyl ring planes. As a consequence, the two pyridyl rings of tpada are not equivalent upon coordination, whereas the simple dpaH ligand without extended $\pi$-conjugation to a carbonyl at the central amine retains an equivalency of the coordinated pyridyl rings on a dpaH ligand as discussed for the [$RuCl_3$(NO)(dpaH)] complex in a prior section of this paper. The $^{13}C$ NMR spectra for free tpada and coordinated tpada of the 2 : 1 $RuCl_3(NO)$ : tpada complex exhibit the anticipated changes upon coordination, retaining equivalency for positions, even those differentiated by $^1H$ NMR methods. A spectrum of the 2 : 1 complex and the free ligand could not be directly compared. The solubility of free tpada is very low. In order to obtain a sufficiently high concentration that would allow for $^{13}C$ data collection, we had to protonate the tpada as $H_2tpada^{2+}$. In this species the protons are not equivalently shared by the pyridyl N donors, causing inequivalency of all but the C-2,2' carbons. Upon coordination, the equal donation of each N-donor of the bidentate headgroup of tpada toward $RuCl_3(NO)$ restores the equivalency of the pairwise assignments for the two pyridyl rings. Thus, the H-6,6' protons are more sensitive to differences within the whole complex, stemming from the electronic withdrawing effect and the nearness to the $\pi$-conjugation of the amide system than are the pyridyl ring carbons whose energies are dominated by their position within the pyridyl $\pi$-cloud rather than peripheral electron densities. The location of the chemical shifts for the ring carbons in the {[$RuCl_3$(NO)]$_2$(tpada)} complex are in general upfield by ca. 1 ppm of those of the protonated $H_2tpada^{2+}$ species.

The infrared spectra of the tpada ligand and its 2 : 1 complex reveal added features concerning coordination. The nitrosyl stretching frequency is at 1872 $cm^{-1}$ in {[$RuCl_3$(NO)]$_2$(tpada)} compared to 1850 $cm^{-1}$ in [$RuCl_3$(NO)(dpaH)], indicative of the fact that the withdrawing amido functionality of tpada allows for weakened back-donation of the $Ru^{II}$ center to coordinated $NO^+$. The free tpada with back-donation from $Ru^{II}$ into the pyridyl ring- amido assembly has weakened carbonyl bonds as exhibited by amide stretches at 1655 and 1604 $cm^{-1}$ in the complex compared to the stronger carbonyl bonds with strettches at 1679 and 1585 $cm^{-1}$ in the starting tpada ligand.

### *[Ru(NO)Cl(pida)] Complex (5)*

The $^1H$ NMR spectrum of the free ligand $pida^{2-}$ and its complexes derived from (**1**) are shown in figure 1A and 1B, repectively. The ligand resonances are defined on the figure. The $CH_2$'s of the glycinato arms appear as a singlet, being freely rotating groups in $D_2O$ solution. Ring protons for H-6 is an anticipated doublet at 8.60 ppm, H-4 is a multiplet at 7.91 ppm and overlapped H-3 and H-5 protons resonating from 7.46 to 7.61ppm.There are several isomers in the aboundance of 64%, 36% and ca. 1% formed when $pida^{2-}$ coordinates to (**1**). These are identified as isomers A, B and C in the figure.

The major isomer A has resonances at 8.71 ppm (doublet) for H-6, 8.25 ppm (multiplet) for H-4 and 7.86 ppm for the H-3, H-5 pair. The $(CH_2)$py protons are present in a coordinated chelate as shown by the AB pattern at 5.38 and 5.03 ppm ($J_{AB}$ = 15.8 Hz). Two equivalent $CH_2CO_2^-$ methylenes have another AB pattern at 4.65 for $H_A$ and 4.44 ppm for $H_B$. This requires carboxylate chelation and

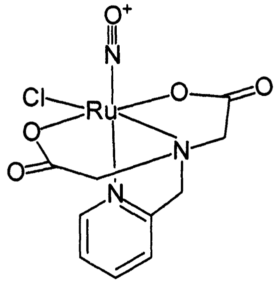

8

a symmetrical placement of those donors. This defines the structure as shown here in 8 for isomer A.

Further evidence for this structure of the dominant species was obtained from a 1676 $cm^{-1}$ carboxylate stretch indicating coordinated carboxylates) and a nitrosyl stretching frequency of 1889 $cm^{-1}$.This $\upsilon_{NO}$ value requires the influence of a "trans strengthening" axial donor as has been described in the literature [17 - 20]. From this, we infer that the pyridyl donor of $pida^{2-}$ is trans to $NO^+$ in isomer A, consistent with the placement of the two carboxylate donors at "in-plane" equivalent locations as in 8 above. Since the sample is a mixture of 64% isomer A and 36% isomer B in which a component to the band would appear near 1871 $cm^{-1}$ for isomer B's contribution, the true nitrosyl stretching frequency for isomer A is calculated as 1898 $cm^{-1}$ from weighted averages.

The lesser isomer B has the H-6 resonance at 8.88 ppm (doublet), H-4 at 8.33 ppm (multiplet, and the H-3, H-5 pair at 7.88 and 7.70 ppm. An AB pattern for the lesser isomer's $(CH_2)$py features are located at 4.56 ppm and > 4.69 ppm, the latter being hidden by the HOD resonance. A singlet of area matching the corrected integration of the $(CH_2)$py group is found for isomer B at 3.78 ppm as a singlet. This implies a pendant glycinato arm. Since there are no other resonances that integrate for the remaining glycinato functionality, we are forced to assume that it is coordinated and buried by the HOD resonance as would be expected for a more downfield resonance, caused by less cancellation of charge at the $Ru^{II}$ center because of a pendant carboxylate. The differences in the pyridyl region from the trans donor in isomer A implicates that the pyridyl group is placed cis to $NO^+$ in isomer B as shown in drawing 9. In the earlier estimation of the nitrosyl stretching frequency for isomer A, we assumed a value for $\upsilon_{NO}$ close to 1871 $cm^{-1}$, the value for the $[RuCl_3(NO)(imidazole)(H_2O)]$ complex wherein the one N-heterocyclic donor is cis to $NO^+$ [7].

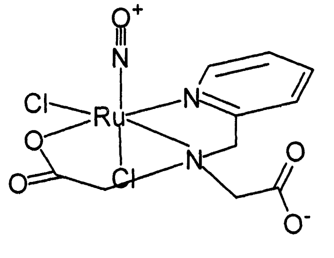

9

The very low abundance isomer C has pyridyl resonances at about 8.22 ppm, from 8.25 to 8.33 and as a shoulder on the more major isomers at 7.69 ppm. There are no simple singlets for pendant carboxylates and the locations of the coordinated $CH_2CO_2^-$ resonances for C will be split beyond detection under the more major resonances. We conclude that isomer C is most probably one in which the pendant carboxylate of isomer B has displaced a $Cl^-$ and is bound as in drawing 10 ; in any event, the amount of isomer C is very small. We have included it for completeness, and because its presence is required to account for accurate integration areas for some of the $CH_2$ and pyridyl resonances of isomers A and B.

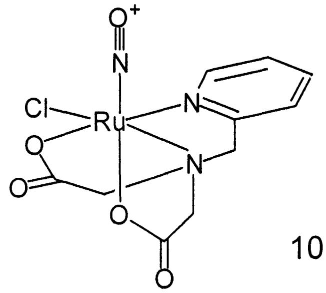

10

All of the observations reported from the $^1$H NMR and infrared data are confimed by the $^{13}$C NMR data on (**5**). In the free ligand $^{13}$C NMR spectrum of the parent pida$^{2-}$ ion, the carboxylate carbons resonate at 170.46 ppm. The methylene region shows one resonance at 58.82 ppm of twice the intensity of another at 56.88 ppm. These are assigned to the glycinato methylenes and the pyridyl arm methylene, respectively. Upon coordination of pida$^{2-}$ to (**1**), the $^{13}$C NMR spectrum shows multiple sets for each C resonance that support the presence of the three isomers (A, B, C) as discussed above from $^1$H NMR assignments. Some of the coordinated resonances are shifted as much as 17 ppm. Three types of carboxylate carbons, two coordinated with shifts of 181.55 (downfield by + 11.10 ppm of the free L) and 179.81 ppm (downfield by +9.36 ppm) appear for isomers A and B, respectively. A resonance for the pendant carboxylate of isomer B is at 174.02 ppm. Resonance for the isomer at 1% abundance according to the $^1$H NMR results are too weak for carbonyl carbons for detection.

The carbons in the pyridyl ring are shown with shifted resonances in two separate sets for isomers A and B. Since these protons have a C-H bond and give greater intensity than for carbonyl carbons, even the 1% isomer C is detected slightly above the baseline noise. The major isomer A has resonances at 159.94 ppm (downfield by +10.47 ppm of the free L) for C-2, at 152.64 ppm (downfield by +9.36 ppm) for C-6, at 145.11 (downfield by +6.37 ppm) for C-4, at 142.41 ppm for C-3 (downfield by +17.08 ppm) and at 125.81 (downfield by +0.87 ppm) for C-5.

Supporting the presence of a second isomer B are the pyridyl resonances at 160.91 ppm (+11.44 ppm downfield) for C-2, at 151.29 ppm (+2.26 ppm downfield) for C-6, at 143.35 ppm (+ 4.61 ppm downfield) for C-4, at 127.45 ppm (+2.12 ppm downfield) for C-3 and at 125.52 (+0.58 ppm downfield) for C-5. The methylene carbons also exhibit downfield shifts as a a result of coordination. These are identified by five resonances, indicative of two isomers-- one with equivalent glycinato arms and apyridyl methylene, and another with three different methylenes which requires one coordinated carboxylate and one pendant carboxylate and the associated pyridyl methylene.

The equivalent glycinato methylenes for isomer A resonate at 73.48 ppm (downfield by + 14.66 ppm of the free L). The matching pyridyl methylene is detected at 65.89 ppm (downfield by +9.02 ppm). Those for isomer B are detected at 70.02 ppm (downfield by +11.20 ppm) for the coordinated glycinato arm, at 55.98 ppm (*upfield* by - 2.84 ppm) for the pendant glycinato arm and at 56.13 ppm (*upfield* by -0.74 ppm) for the pyridyl methylene of isomer B.

### Discussion

The $^1$H and $^{13}$C NMR data for $[RuCl_3(NO)(dpaH)]$ = (**2**) establish the coordination and non-lability of the dpaH ligand in this complex. The nitrosyl stretching frequency at 1850 $cm^{-1}$, below the 1895 $cm^{-1}$ stretch for (**1**), shows that coordination produces a weakening of the $NO^+$ stretching frequency, rather than the "trans strengthening" when a stronger donor than $Cl^-$ is trans to $NO^+$ [17-22]. This confirms the "in-plane" coordination of dpaH, the *fac* placement of the three $Cl^-$ donors, and the NMR equivalency of the two pyridyl donors.

When the second dpaH ligand is added to (**2**) forming $[Ru(NO)Cl(dpaH)_2]Cl_2$ = (**3**) in the isolated solid, both dpaH ligands are retained in the plane cis to $NO^+$ as shown by the NMR equvalency of the four pyridyl donors, and the absence of change in the $\upsilon_{NO}$ value (again 1850 $cm^{-1}$ for the bis complex). By contrast, the bis bipyridine complex of seeming identical inner-sphere coordination with bpy replacing dpaH, $[Ru(NO)Cl(bpy)_2]^{2+}$, exhibits the nitrosyl stretch at 1931 $cm^{-1}$. This is an example of the "trans strengthening" effect as one of the pyridyl donors of bipyridine must be placed in the axial site trans to $NO^+$ in order to avoid steric contacts that occur for two in-plane bpy ligands. The flexibility of the dpaH ligand around the central amine NH affords adjustability in the case of dpaH, such that two in-plane donors can coordinate, yet avoid the steric difficulties of the more planar bpy system. Models have been built which clearly show that a flexing motion can be achieved that place the H-6,6' hydrogens of each dpaH ring set in (**3**) above the $RuN_4$ plane and close to $NO^+$ for 50 percent of the time, while the alternate dpaH donor projects its H-6,6' set below the $RuN_4$ plane. The flexing motion of the rings allows for equilibration of the two configurations, and hence NMR equivalency.

The very similar UV-visible electronic spectra for complex (**1**), (**2**) and (**3**) emphasizes the fact that the observed electronic transition near 430 nm and 435 nm for (**2**) and (**3**), respectively, like those in the cyclam analogue of (**3**) [16], are $Ru^{II}$ to $NO^+$ MLCT transitions in character admixed with a d-d component. The dominance of the MLCT nature accounts for extinction coefficients above 10 $M^{-1}cm^{-1}$ and the relative insensitivity to the cis in-plane donors.

The formation of the binuclear complex {$[RuCl_3(NO)]_2$(tpada)} = (**4**) shows that a more competitive π-acceptor ligand in the in-plane position can effectively compete with $NO^+$ for back-donation. In doing this, the back-donation to $NO^+$ decreases and the nitrosyl stretching frequency increases from the 1850 $cm^{-1}$ value with one dpaH ligand in (**2**) to the observed value of 1872 $cm^{-1}$ in (**4**). The effect is not large enough to achieve values above 1895 $cm^{-1}$ that would be characteristic of a trans placement of one pyridyl donor. Hence, the coordination of the tpada headgroup in the binuclear complex (**4**) is still in the cis-to-$NO^+$ arrangement as in the simple dpaH complex (**2**). The present study has shown that it is possible to prepare a binuclear tethered "caged NO " reagent that is structurally capable of spanning the major groove of DNA and afford covalent bond formation by displacement of a $Cl^-$ donor at the Ru center. Solubility difficulties with the binuclear complex (**4**) cause this complex, itself, to have limited potential use. It is desirable to prepare $\{Ru(NO)\}^6$-chelated and tethered complexes wherein the chelating site is a π-donor rather than the π-acceptor competitor ligand as in tpada. However, the binuclear complex (**4**) is important as a working model for the design of better antitumor metallodrugs that carry forth the present theme. The better π-donating peptido complex of $\{Ru(NO)\}^6$ derived from (gly-gly-gly-2H) [8] hold high promise in this regard, combining bio-compatibility of the ligand with ones that can be incorporated with more soluble spacer ligand components via rather common synthetic strategies.

The [Ru(NO)Cl(pida)] complex = (**5**) occurs as several isomers. The major isomer (64%) has the pyridyl donor in the position trans to $NO^+$, causing a trans strengthening of the nitrosyl stretch at a calculated value of 1898 $cm^{-1}$. An alternate placement of the pyridyl donor at an in-plane position cis to $NO^+$ occurs 36 % of the time. In this arrangement, strain and the less good trade of an axial carboxylate for $Cl^-$ than a pyridyl donor for $Cl^-$ prompts a pendant carboxylate instead of the full quaternary donation available from $pida^{2-}$ that is displayed in the major isomer. In a prior study with the more simple N-methyliminodiacetate ligand ($mida^{2-}$) that lacks the pyridyl donor of $pida^{2-}$, we observed that one glycinato donor of the two available in $mida^{2-}$ achieves in-plane coordination to $RuCl_3(NO)$ and one coordinates axially with the amine donor cis to $NO^+$ [7]. The observed AB pattern with $H_A$ = 4.71 and $H_B$ = 4.04 is similar to the in-plane values of $H_A$ = 4.68 and $H_B$ = 4.40 whereas axial coordination of a glycinato methylene has resonances at $H_A$ = 4.31 and $H_B$ = 3.89, less downfield than for the in-plane glycinato chelation. Alteration of synthetic conditions, kinetic influences, and the orientational placement of donor groups along the synthetic pathways appear to be crucial in establishing $\{Ru(NO)\}^6$ bonds to carboxylate donors because both are coordinated in the major isomer (64%), and the least abundant isomer (1%), but not the second most abundant isomer (36%). For synthetic purposes, a correct choice of conditions might favor a unique distribution. But finding such a system was not a goal of the present work. Rather, it is deemed of greater value to determine what kind of ligand sets make stable carriers for the $\{Ru(NO)\}^6$ chromophore. Simple monodentate imidazole donors are insufficient to produce non-labile complexes [7]. Chelation at least as extensive as bidentate N donors (as in dpaH) is essential for non-lability, and the potentially tridentate $mida^{2-}$ ligand is also too labile. Inclusion of a second N donor as in $pida^{2-}$ yields a stable system for $RuCl_3(NO)$-based complexes.The neutral complex such as the $pida^{2-}$ complex in (**5**) may be useful as a transporter molecule to get "caged NO " across cell walls. Further work in this regard, and the photo-reactivity studies of these materials are in progress.

Since tumor cells are known to concentrate ruthenium chloro, ammine, and polyaminopolycarboxylate complexes [23 - 26], and the $RuCl_3(NO)$ chromophore is related as a simple chloro ruthenium complex to the imidazolium salt, [imH]{trans-$[RuCl_4(im)_2]$} in use against colorectal and solid tumors [27 - 30], the advances herein contribute to the possible antitumor therapies for a number of tumor cell lines. The complexation of the $RuCl_3(NO)$ core by aminocarboxylate donors as reported previously [6 - 8] and in the present manuscript are of value in the design of peptide-transportable antitumor agents. The advantage for those derived from (**1**) resides in their control against redox side reactions during the transport phase and the possibility of "turning them on" by selective photolysis, once the species is absorbed by the intended tumor.

**Acknowledgment**

The authors gratefully acknowledge the support of the Petroleum Research Fund, administered by the American Chemical Society, for this work.

**References**

1. N. Bettache, T. Carter, J. E. Corrie and D. Ogden, *Methods in Enzymology,* **268**, 266 (1996).
2. T. D. Carter, N. Bettache and D. Ogden, *Brit. J. Pharmacol.,* **122**, 971 (1997).
3.P. C. Ford, J. Bourassa, K. Miranda, B. Lee, I. Lorkovic, S. Boggs, S. Kudo and L. Laverman, *Coord. Chem. Rev.,* **171**, 185 (1998).
4. G. Stochel, A. Wanat, E. Kulis and Z. Stasicka, *Coord. Chem. Rev.,* **171**, 203 (1998).
5. T. Ishiyama, T. Matsumura and Y. Honda, *Radioisotopes*, **30**, 361 (1981).
6. T. A. Balakaeva, A. V. Churakov, M. G. Ezernitskaya, L. G. Kuz'mina. B. V. Lokshin and I. Efimenko, *Russ. J. Coord. Chem.,* **25**, 579 (1999).
7. J. M. Slocik, M. S. Ward and R. E. Shepherd, *Transition Met. Chem. (London),* **25** (2000), submitted.
8. J. M. Slocik and R. E. Shepherd, *Inorg. Chim. Acta,* (2000), submitted.
9. J. B. Godwin and T. J. Meyer, *Inorg. Chem.,* **10**, 471 (1971).
10. R. E. Shepherd, Y. Chen, R. A. Kortes and M. S. Ward, *Inorg. Chim. Acta,* (2000), in press.
11. J. M. Fletcher, I. L. Jenkins and F. M. Lever, *J. Inorg. Nucl. Chem.,* **1**, 378 (1955).
12. R. E. Shepherd, M. A. Sweetland and D. Junker, *J. Inorg. Biochem.,* **53,** 1 (1997).
13. Y. Chen, F.-T. Lin and R. E. Shepherd, *Inorg. Chem.,* **38**, 973 (1999).
14. Y. Chen, F.-T. Lin and R. E. Shepherd, *Inorg. Chem.,* **36**, 818 (1997).
15. Y. Chen, F.-T. Lin and R. E. Shepherd, *Inorg. Chim. Acta,* **268**, 287 (1998).
16. D. R. Long, J. A. Davis, L. G. F. Lopes, A. A. Ferro, L. C. G. Vasconcellos, A. Wieraszko and M. J. Clarke, *Inorg. Chem.,* (2000), in press.
17. H. Tomizawa, E. Miki, K. Mizumachi and T. Ishimori, *Bull. Chem. Soc. Jpn.,* **67**, 1809 (1994).
18. H. Tomizawa, E. Miki, K. Mizumachi and T. Ishimori, *Bull. Chem. Soc. Jpn.,* **67**, 1816 (1994).
19. Y. Suzuki, H. Tomizawa and E. Miki, *Inorg. Chim. Acta,* **290**, 36 (1999).
20. H. Tomizawa, K. Harada, E. Miki, K. Mizumachi, T. Ishimori, A. Urushiyama and M. Nakahara, *Bull. Chem. Soc. Jpn.,* **66**, 1658.
21. A. A. Batista, C. Pereira, S. L. Queiroz, L. A. A. Olivera, R. H. deA Santos and M. T. doP. Gambardella, *Polyhedron,* **16**, 927 (1997).
22. S. Dhf, O. G. Teixeira, and A. A. Batista, *Polyhedron,* **14**, 1031 (1995).
23. G. Sava, S. Pacor, F. Bregant and V. Ceschia, *Anticancer,* **11**, 1103 (1991).
24. M. J. Clarke, T. C. Zhu and D. R. Frasca, *Chem. Rev.,* **99**, 2511 (1999).
25(a). M. J. Clarke in *Platinum, Gold, and Other Chemotherapeautic Agents,* S. J. Lippard, ed., ACS, Washington, D.C., **209**, 335 (1983).(b) M. J. Clarke in *Metal Ions in Biological Systems,* H. Sigel, ed., Dekker : New York, **11**, 231 (1980).
26. R. E. Shepherd, Y. Chen, S. Zhang, F.-T. Lin and R. A. Kortes, *Adv. Chem. Ser.,* **253**, 367 (1997).
27.(a) W. Petri, T. Pieper, M. Sommer, B. K. Keppler and G. Geister, *Eur. J. Inorg. Chem.,* **9**, 155(1999). (b)B. K. Keppler, W. Rupp, U. W. Juhl, H. Endres, R. Niebel and W. Balzer, *Inorg. Chem.,* **26**, 4366 (1987).
28.(a) B. K. Keppler and W. Rupp, *J. Cancer Res. Clin. Oncol.*, **111**, 166 (1986). (b) A. Galeano, M. R. Berger and B. K. Keppler, *Arzneimittel-Forsch,* **42**, 821 (1992).
29. B. K. Keppler, M. R. Berger and M. E. Heim, *Cancer Treatment Review,* **17**, 261 (1990).
30.(a) F. Kratz, B. K. Keppler, L. Messori and E. N. Baker, *Metal-Based Drugs,* **1**, 169 (1994). (b) J. Chatlas, R. van Eldik and B. K. Keppler, *Inorg. Chim. Acta,* **233**, 59 (1995).

**Received: February 21, 2000 - Accepted: February 10, 2000 - Received in revised camera-ready format: February 12, 2000**

